# Are some cultures more mind-minded in their moral judgements than others?

**DOI:** 10.1098/rstb.2020.0288

**Published:** 2021-11-22

**Authors:** H. Clark Barrett, Rebecca R. Saxe

**Affiliations:** ^1^ Department of Anthropology and Center for Behavior, Evolution, and Culture, University of California, Los Angeles, Los Angeles, CA, USA; ^2^ Department of Brain and Cognitive Sciences and McGovern Institute for Brain Research, Massachusetts Institute of Technology, Cambridge, MA, USA

**Keywords:** cooperation, morality, theory of mind, strict liability, mental opacity, cultural main effects

## Abstract

Cross-cultural research on moral reasoning has brought to the fore the question of whether moral judgements always turn on inferences about the mental states of others. Formal legal systems for assigning blame and punishment typically make fine-grained distinctions about mental states, as illustrated by the concept of *mens rea*, and experimental studies in the USA and elsewhere suggest everyday moral judgements also make use of such distinctions. On the other hand, anthropologists have suggested that some societies have a morality that is disregarding of mental states, and have marshalled ethnographic and experimental evidence in support of this claim. Here, we argue against the claim that some societies are simply less ‘mind-minded’ than others about morality. In place of this cultural main effects hypothesis about the role of mindreading in morality, we propose a contextual variability view in which the role of mental states in moral judgement depends on the context and the reasons for judgement. On this view, which mental states are or are not relevant for a judgement is context-specific, and what appear to be cultural main effects are better explained by culture-by-context interactions.

This article is part of the theme issue ‘The language of cooperation: reputation and honest signalling’.

## 1. Mind-mindedness in moral judgements across cultures

For many people steeped in ‘Western’ moral, legal and philosophical traditions, it may seem a foregone conclusion that moral judgements are fundamentally about individuals' intentions, motivations, beliefs, desires and character. When one person harms another, laypeople and legal traditions in the USA and Europe take into account the person's *reasons* for the action: discriminating harms done for ‘good’ reasons (e.g. punishment, self-defence) from those done for ‘bad’ reasons (e.g. selfish exploitation), and discriminating harms done uncontrollably or inadvertently (accidents, mistakes) from harms done purposefully with specific intent [1–7].

Yet, the pattern of moral judgements in European cultures and their colonial derivatives may not be representative of human ways of thinking. Does the consideration of individual mental states for moral judgement vary across different cultural groups? In particular, are there places where people take into account only observable actions and outcomes, rather than *reasons* for those actions when assigning blame and punishment? It is clearly theoretically possible that such cultural groups could exist. As described below, for people everywhere, there are some contexts in which people judge others based only on observable actions and outcomes, so there could be societies in which this strategy was applied to all contexts of moral judgements. Theoretically, evolutionary models show that cooperation in social groups can be stabilized without taking others' mental states into account at all, by conditioning cooperation and punishment decisions on others’ publicly observable actions and the outcomes of those actions, not the reasons for them [8,9]. Thus, for a stable, functioning system of moral evaluation, it is not necessary to evaluate individual mental states. The question is whether, as it is not necessary, some groups do not.

People clearly do differ in how they assign blame and punishment. Ethnographic and cross-cultural experimental studies around the world reveal variability in when and how people incorporate evidence of an individual's beliefs and intentions into moral judgements. We will review this evidence, and how it has been theorized, below. Our main question is: what is the source and structure of this variability? One kind of view, which we call the *cultural main effect* (CME) hypothesis, is that cultural groups vary in the degree to which they use mental states for moral judgement. That is, there is a dimension or parameter of cultural variation, distinguishing cultural groups from one another, that determines how mind-minded moral judgements are across the board in each culture. Here, we review evidence and arguments for this claim and conclude that there is no evidence for mental-state-disregarding cultural groups.

By contrast, we argue that people everywhere are sometimes mind-minded in their moral judgements, and sometimes not. Within every society so far investigated, there is variability across contexts, with reasons for actions taken into account more in some contexts than in others. The role of mental states in moral judgement, even within ‘Western’ moral, legal and philosophical traditions, depends on how situations are appraised and for what reasons judgements are being made. Furthermore, there are shared patterns across cultural groups, between contexts that attract more or less mind-minded moral judgements. By considering this contextual variation, we stand to gain a more accurate picture of human morality than by ranking cultural groups on mind-mindedness as a whole.

## 2. The evidence for cultural differences in mindreading

It is well established that consideration of an individual's mental state, as in the concept of *mens rea,* plays a central role in many legal systems [1]. Moreover, a considerable body of psychological research, done largely in the USA and Europe, shows that people in these places do indeed sometimes condition their moral judgements of others on judgements about the reasons for their behaviour, including intentions, motivations and knowledge states that are deemed relevant for assigning guilt and blame [2–6]. This literature shows that people's moral judgements can make use of very flexible, sophisticated and fine-grained distinctions between mental states which generate culpability, relying on the evolved capacity known as theory of mind [10]. Indeed, intent-based moral judgement seems to develop by pre-school years, at least in these societies [9,11].

The anthropology literature suggests that this pattern might not be universal. Multiple lines of evidence suggest that there is variability, across cultural groups, in how mental states are incorporated into moral judgements.

### (a) Ethnographic evidence

Ethnographers have long noted that punishment for serious violations does not necessarily depend on the accused person's mental state. If one person causes an injury or a death, or damages someone else's property, in many societies, the victim or his family may demand compensation or extract revenge, without considering the offender's reasons, or whether the harm was caused intentionally or accidentally [12,13]. Indeed, often the whole families of the victim and offender become involved, either in negotiating and paying compensation or in a cycle of revenge, even if most members of the family were completely unaware of the original offence.

Many ethnographers have noted that social groups have procedures for dealing with homicide, by punishment and/or compensation, that does not distinguish between intentional and accidental causes of death. In many societies with so-called honour killing or ‘blood revenge’, if a person from one family kills a person from another family, it does not matter whether the killing was accidental, deliberate or even in self-defence; the victim's family is entitled to seek revenge in kind [14,15].

To collect and analyse such ethnographic observations, Curtin *et al.* [16] used Human Relations Area Files (HRAF), an archive of historical ethnographies organized to facilitate coding of cultural traits for comparative analyses. Curtin *et al.* [16] searched the database of 146 societies for evidence of ‘strict liability’—the same punishment or compensation following a harm, usually death—as well as ‘collective guilt’, which also typically downplays the intentions or motives of single individuals. The concept of strict liability, in Western legal systems, is that a defendant is held responsible for the consequences of their actions, regardless of whether those consequences were desired or even foreseen by the defendant [17]. In HRAF, of 38 societies for which relevant information was available, 16 (42%) were classified as having some strict liability norms (typically, for homicide). For collective guilt, 45 of the 71 (63%) societies that could be coded provided evidence of it.

Another ethnographic case of moral judgements that do not distinguish between intentional and accidental harms comes from Malagasy judgements about incest. In many regions of Madagascar, sexual unions between proscribed categories of kin (incest) are believed to cause widespread catastrophe: failed crops, accidents, miscarriages and child deaths. Astuti & Bloch [18] show that the consequences of incest are believed to unfold regardless of whether the people who committed the incest did so knowingly or not. Malagasy informants explain that considerations of intentionality are irrelevant: harm occurs whether or not people know they are violating the taboo, it befalls other parties as well, and many innocent people must pay the costs of ritual reparation for the harm.

Ethnographers working in Micronesia and Melanesia have described an even broader disavowal of individual mental states, as a ‘norm of mental opacity’ [19–22]. Informants, in these ethnographies, explicitly state that it is either impossible or undesirable to infer the mental states of others. Parents discourage children from referring to others' thoughts and feelings, and direct their attention instead to visible actions (e.g. crying, not sadness, [16]). In ethnographic fieldwork among the Ku Waru people of highland Papua New Guinea, Rumsey [21] found that people assert that other minds are opaque and unknowable. Rumsey [21] analysed transcripts of informal parent–child conversations and formal legal arguments. In their conversations, Ku Waru parents and young children spontaneously refer to specific beliefs or desires much less frequently than comparable pairs of North American parents and children. In the transcripts of legal disputes, Ku Waru people almost never ascribe a specific thought to another person, and instead, explicitly argued that a person's reasons for committing a violation could be known only to God.

### (b) Cross-cultural experimental evidence

To complement the observations of ethnographers, cross-cultural experiments have asked people in different places to assign blame and punishment to characters in the same hypothetical vignettes. The CME hypothesis predicts that there is substantial variation in the role of mental states in these moral judgements; and specifically, that this variation is between social groups.

To investigate the effect of a norm of mental opacity on people's moral judgements, McNamara *et al*. [23] compared Indigenous iTaukei Fijians on Yasawa Island with Indo-Fijians and a sample of Canadians and Americans on a series of moral judgement tasks comparing accidental harms, failed attempts and intentional harms. iTaukei were more outcome-oriented than Indo-Fijians and North Americans, judging accidental harms to be morally worse. The experimenters also experimentally manipulated focus on either the character's thoughts or their actions, using a priming paradigm. The iTaukei Yasawa Islanders were more influenced by the ‘thought’ prime than people from the other groups—suggesting, perhaps, that their default was not to attend to others' thoughts in judging their actions.

The largest systematic cross-cultural experimental study of the role of mental states in moral judgement was conducted by Barrett *et al*. [24]. Sampling 10 different societies, this study examined multiple kinds of moral violations including battery, theft, poisoning of a well and eating proscribed food. It also examined multiple mental states underlying these actions, including intentional commission, accident, mistake of fact, insanity, self-defence, necessity and different moral beliefs. For example, the ‘battery’ scenario concerned a man in a crowded market place who punched another man in the face, either in self-defence (the other man attacked with a knife), out of necessity (the other man was obstructing the path to douse a dangerous fire), or based on a reasonable mistake of fact (falsely believing that his son was being attacked). The theft scenario concerned a man who took someone else's similar-looking bag from the market place, on purpose or by mistake. The poisoning scenario concerned a man who put insecticide in a local water source, either knowing that it was poisonous or believing that it was safe.

Barrett *et al*. found substantial variation across cultures in how people conditioned their moral judgements on actors' mental states. Most dramatically, people in Western, educated, industrialized, rich, democratic societies (e.g. in Los Angeles) accepted justified mistakes as basically completely exonerating of battery, poisoning and theft, but people in most other societies did not. In some societies, people judged the man who poisoned the well or punched the stranger based on a false belief to be nearly or exactly as bad as if he had caused the harm knowingly and purposefully. In a re-analysis of the same data, Curtin *et al*. [16] found that the average exoneration by mistakes and accidents was negatively correlated with a society's kinship intensity. That is, people from societies with tight kinship exonerate mistakes less than do people from societies with loose kinship.

In summary, both ethnographic fieldwork and experimental data show that people sometimes disregard a person's mental state when judging them to be blameworthy and/or to deserve punishment for causing harm or violating a norm. Meaningful variance between people is explained by the population or society from which the person was sampled; the same scenario receives different moral judgements from people in different places. Anthropologists and psychologists have suggested that these differences may reflect broad differences in how people from different societies think about minds. In particular, the CME hypothesis suggests that cultural groups differ from one another in the overall weight given to mental states in moral judgements. In the next section, we consider this hypothesis in more detail.

## 3. Theoretical variants of the cultural main effect hypothesis

Why would people from some cultural groups, as a whole, be less mind-minded about moral judgements? The CME is a descriptive hypothesis about the structure of variance in people's judgements. Yet at the same time, the CME hypothesis is often supported by more speculative arguments about the cause of this variation. We can distinguish variants of the CME hypothesis that offer different interpretations of why cultural groups differ in this way. Specifically, the CME hypothesis could hold that people in some societies (i) will not, (ii) should not, or (iii) cannot consider mental states when making moral judgements.

The first variant of the CME hypothesis (will not) suggests that people in some societies could consider individual mental states for moral judgement, but they are unlikely to do so in practice because there is insufficient benefit to doing so. Considering an individual's beliefs and desires would be pointless if, for example, the context is so constraining that individuals never get to choose their course of action. If the only food available is rice, then eating rice provides no evidence of the eater's desires. If life in general, for a whole cultural group, was so constrained that no one made individual choices, then people in that group might not tend to consider individuals' beliefs and desires when predicting, or explaining, actions. Thus, for example, McNamara *et al*. [23, p. 104] argue that ‘tight behavioural structuring reduces [the] informational value of mental states’: in societies with rigidly defined social roles which guide behaviour, little predictive power is gained by considering the individual's motivations and intentions.

This first variant of the CME hypothesis may be accompanied by corollary hypotheses about the nature and function of theory of mind. First, the nature of theory of mind: the plausibility of this variant of the CME hypothesis depends on how effortful it is for people to consider others' mental states. If theory of mind is cognitively costly to deploy, then people would refrain from using it unless the benefits are large and frequent. By contrast, if theory of mind is relatively effortless to deploy, then even relatively small or infrequent benefits would be enough to sustain people's probability of using it. Second, the function of theory of mind: some theorists have suggested that considering an individual's mental states is most useful when choosing interaction partners, versus when interacting with existing partners [25,26]. If you can never switch jobs, it might feel pointless to evaluate the leadership style of your current boss. If so, then engaging in theory of mind would be have little value in a cultural group where people are rarely able to choose with whom they interact.

The second variant of the CME hypothesis (should not) suggests that people in some societies believe it is inappropriate, rude or otherwise undesirable to consider others' mental states. This is one potential interpretation of norms of mental opacity. The insides of other minds could be treated like private territory, not to be broached from the outside. People with this belief would try to refrain from, or even suppress, theory of mind. By contrast to the earlier picture of theory of mind as cognitively costly to deploy, this view implies that theory of mind is spontaneously active and requires effort or training to suppress.

The third variant of the CME hypothesis (cannot) suggests that in some societies, people cannot consider individual mental states for moral judgement, because they are missing a ‘cognitive gadget’ for that way of thinking [27]. Mindreading could be a culturally invented cognitive gadget, analogous to a bicycle: for people raised riding bicycles, it is a skill that becomes effortless, yet bicycles do not exist everywhere. Using mental states in moral judgement could be an analogous cultural invention. Children might learn from adults' explicit teaching that what matters when one person harms another, is to consider the person's reasons. Sufficient experience might make this skill effortless, but people not exposed to it would never develop it. If mind-mindedness is a gadget in this sense, then people who have not been taught theory of mind cannot make mind-minded moral judgements.

In summary, the CME hypothesis that mind-mindedness of moral judgements varies across societies can be interpreted in different ways. What these hypotheses have in common is that the variation is between societies: a whole group of people puts less weight on mental states, for all moral judgements, and possibly for social life more generally. In the next section, we argue that the evidence does not support this view.

## 4. Arguments against the cultural main effect hypothesis

The evidence summarized in §2 may seem like strong support for cultural differences in mindreading. Here, we argue that what in each case may seem like a CME is plausibly a much narrower difference in how a specific type of mental state is incorporated in a specific type of moral judgement. The striking variation in the role of mental states in moral judgement occurs not as a context-general effect across societies, but within societies across contexts. In societies that have been described as ‘low’ in mind-mindedness, there are contexts in which mental states are considered in moral judgements; and in societies that have been described as ‘high’ in mind-mindedness, there are contexts in which mental states are not considered in moral judgements.

The examples where mental states are not considered seem to be special cases, within societies. In the case of strict liability, while Curtin *et al*. [16] characterize whole societies as either governed by strict liability norms or not, the evidence is in fact more consistent with a more limited effect. In most of the societies they consider, rules for compensating damages recognize the difference between accidents and intentional harms, but restitution rules are designed to make victims whole. For example, Curtin *et al.* classify the Igbo as an ‘intermediate’ case of strict liability, but Elechi states simply, ‘Igbos distinguish between murder and manslaughter’, going on to describe how punishment and restitution are matters of negotiation that depend on the circumstances of the death and the relationships and histories of those involved, not merely strict liability [28, p. 408].

Outside the limited context of murder, there is no evidence that any of the societies described have strict liability norms of moral judgement across the board. Indeed, given its seriousness, there are good reasons to think that murder might be a special case. For example, while Gikuyu people are described as having strict liability about murder in Curtin *et al*.'s taxonomy, the Gikuyu moral system is clearly attuned to personal qualities and mental states. Kinoti [29, p. 16] describes the five ‘virtues’ of this system as ‘honesty (wĩhokeku), generosity (ũtaana), justice (kĩhooto), courage (ũcamba) and temperance (wĩkindĩria)’, and explains how people make fine-grained judgements about others based on these personal, internal qualities.

The same limits apply to the example of *Loza* in Madagascar. If a couple commits incest, catastrophe ensues regardless of the couples' mental states. However, Astuti & Bloch [18] emphasize that incest is unusual in this respect. For other acts of wrong doing, like breaking a neighbour's bucket or killing a neighbour's chicken, the same adults robustly distinguished between accidents (which would be repaired with an apology and restitution) and intentional harms (which would lead to a village assembly proceeding and punitive damages).

Similarly, Barrett *et al*. [24] found substantial differences within societies, between scenarios, in exoneration by intent. The ‘intentions bank’ of their study looked at the role of intentions (intentionally versus accidentally committing an act) in moral judgement, across four contexts: theft, well poisoning, battery and food taboo. The statistical interaction between intentionality and society—a measure of CME—was modest, and accounted for the same amount of variation as the intentions-by-scenario interaction. The scenario-by-society interaction accounted for about twice as much variance, suggesting that contextual variation in judgements within societies is much greater than the average effect of intentions on judgements across societies.

The ‘mitigating factors’ bank used a single context—one man striking another—but varied the man's reasons for doing so, including his beliefs and motivations (e.g. that he was acting to put out a fire). Every society found some reasons for action, including self-defence and necessity, exculpatory. This suggests that no society in the sample disregarded mental states across the board. Instead, context mattered, and different societies treated the same context differently (e.g. in some places, harm caused in the service of self-defence was considered praiseworthy, in others not—but it was exculpatory of blame everywhere). Finally, some contexts led to reduced importance of mental states across most societies—for example, violating a food taboo. Together these findings support the claim that all societies have contextual variation in when mental states matter, and how, for moral judgement.

What about norms of mental opacity? Are there whole societies where people avoid considering others' mental states? Rumsey [21] has compellingly argued that norms of mental opacity are invoked not to suppress consideration of mental states, but to generate skepticism and vigilance about people's statements of intentions. Rumsey argues that attending to, inferring, and distinguishing other's intentions and knowledge is as ubiquitous in the course of everyday life among Ku Waru people as elsewhere. In legal transcripts, people remind one another to be *skeptical* of testimony about reasons and intentions, because one cannot be certain of another's thoughts. Minds are opaque because people may deliberately hide or misrepresent their thoughts and desires, and only an omniscient deity could know what is truly in another's minds or hearts. In parent–child conversations, explicit references to the possibility of lying and deceit (mentalistic concepts) are much more likely in Ku Waru than in comparable North American dyads. Thus, rather than identifying societies that are free from concern with others' mental states, the mental opacity norm may reveal societies that are particularly *vigilant* about others’ mental states [30].

The converse is also true: people from societies that seem ‘highest’ in mind-mindedness also disregard mental states for some moral judgements [31,32]. The United States (US) legal system has extensive rules and case law about strict liability, cases in which people accused of harm cannot claim ignorance as a defence. The scope of strict liability has varied over the history of American law, yet some kinds of harm have always carried strict liability, such as damage or injury caused by a wild animal that belonged to the person. People with no legal training nevertheless seem to find the applications of strict liability intuitive. For example, consistent with laws in many US states, American adults, with no legal training, intuitively ignored intentions when judging violations of laws with an arbitrary threshold (e.g. speed limits, age of consent, school zone distances), compared to violations of laws without an arbitrary threshold (e.g. reckless driving, sexual battery, drug distribution; [33]).

In summary, we argue there is little evidence for places where the norm or default is to ignore other people's mental states in moral judgement, either in the ethnographic or experimental data. Rather, some *domains* or *contexts* of strict liability probably exist in many and perhaps all cultures. What we need to explain is why the same person in a given society might take the motivation of self-defence into account in exonerating someone for battery, but not mistake of fact; or why mistake of fact might be exonerating for theft, but not for eating taboo meat. More generally, we need to explain why people sometimes disregard others' mental states as a function of who they are, what they are doing, and why the judgement is being made—as opposed to why mental states should not matter across the entire moral domain of a community.

## 5. Context effects on mind-mindedness

By contrast to the CME hypothesis, we argue that most of the variation in mind-mindedness is within societies across contexts. Is the contextual variability arbitrary? Could any context elicit moral judgements that depend highly, or not at all, on mental states? Here, we argue that contextual variability is systematic, and the situations that lead to more focus on individual mental states are highly conserved across societies. Common features of who is accused, what they are accused of and why the judgement is being made lead to more or less mind-minded moral judgements across cultures. Critically, these same contextual features predict more or less mind-minded moral judgements by the supposedly maximally mind-minded people in ‘Western’ moral and legal traditions.

### (a) Who they are

Across societies, mental states are likely to matter most when harm is caused by competent, high status, individuals; and least when harm is caused by incompetent, lower status individuals and/or by groups. As an example, consider collective guilt, or corporate responsibility, discussed by Curtin *et al.* [16]. When responsibility for an injury can be attributed to a group or social organization, mental states of individual actors become less important. The accused group is frequently held collectively liable, regardless of whether all or indeed any of the group members intended the injury. As noted above, there are many examples of this pattern in cultural responses to killings: ‘where organizations are the parties to homicide cases, the killer's state of mind when killing is often irrelevant, and accidental killings may trigger sanctions every bit as severe as intentional killings' [34, p. 88].

Curtin *et al*. [16] suggest that this pattern is characteristic of kinship-intensive societies, but the sociologist of law, Donald Black, notes that the same pattern applies in twentieth-century American case law [35]. Companies are held responsible for misfortunes that were not intended or even foreseeable by individual members of the company. Both case law, and intuitive judgements by legally naive American adults, agree that when an injury is the result of business activity, the business is liable for the injury even if it was neither intended nor even reasonably foreseeable, and an individual in the same situation would not be held personally liable. For example, if a truck driver hits a cyclist, American college students assign greater liability if the truck and the trip were being done for business [36]. Black argues these American legal intuitions reflect the reappearance, in a new form, of traditional patterns. While ‘the organization is replacing the family in the compensation’, as ever ‘the liability of groups is [less dependent on intentions] than the liability of individuals' [35, pp. 59–60]. Thus, corporate responsibility reduces the importance of individual actor's mental states, but such group-related blame is not only or typically characteristic of kinship-intensive societies.

The role of intentions may also depend on the relational distance between the person who caused harm, and the observing judge. Some theorists have suggested that observers should care about a person's intentions when those intentions are diagnostic of the person's future behaviour [26], and when the observer can choose whether or not to interact with that person in the future [25]. If a person cannot control the harm they cause, then their intentions are irrelevant for their future value in relationships [26,37]. On this view, intentions should matter most at intermediate relational distances, when future interactions are possible but not inevitable, and less for one-shot interactions with complete strangers and for permanent relationships like that between parents and children. Yet, there is little empirical evidence for a greater role of intentions at intermediate relational distances. We suggest that even when people cannot choose *whether* to interact with the same partner in the future, there remains a lot of scope for choosing *how* to interact with that partner. As a result, substantial consideration of intentions may occur not only at intermediate social distances (because malevolent intentions are informative of a potential partner's character), but also with complete strangers (because malevolent intentions are particularly dangerous) and with long-term committed partners (because malevolent intentions have high emotional stakes).

### (b) What they are doing

Individuals’ mental states matter less when making moral judgements of inherently dangerous or uncontrollable actions. For example, in a series of vignettes about an attempted poisoning, the mental state (desire to poison, belief that it was poison) and outcome (no injury) were held constant. Nevertheless, American adults assigned less punishment, when the substance was actually less dangerous [38]. Another experiment compared judgements of a truck driver who injured a cyclist after unforeseeably hitting a nail in the road; the driver was held more liable when the truck contained dangerous chemicals [36].

People may disagree about whether the actions were inherently dangerous. Should a toymaker have known that paint might contain lead [39]? Should a chemist have known that his factory might release a dangerous fume [36]? Even given the same information about the reasons for these beliefs, people drawn from the same population disagree with each other about the reasonableness of the mistake. There is also substantial disagreement about what counts as reasonable and prudent care. For example, if an accident could have been prevented but the necessary precautions are not common, was the accident negligent or not? American courts reached both answers to this same question in comparable cases [40]. Thus, apparent cultural differences in intent sensitivity may arise from differing appraisals of what counts as reasonable prudence and care in particular situations, and for particular actors [26,41,42]. People who rely on scarce water, for example, may feel that putting any substance in a communal water supply is inherently dangerous so that even ‘accidental’ well poisoning is judged harshly, independent of the character's intentions [24].

Even complete ignorance of the possibility that harm *could* occur is not mitigating if the actor is perceived to have a moral obligation to investigate and find out about the possibility of harm. Discourses about microaggressions in the contemporary USA are a case in point: some people feel that the individual's mental states are exonerating (but it was meant as a compliment!), while others argue that each individual has a responsibility to self-educate about and then avoid the resulting harms to others [43]. Apparent differences in intent sensitivity across cultural groups might thus arise from different perceptions of the specific mistake as reasonable—not from cultural variation in whether reasonable mistakes are exonerating, but from narrower standards about reasonableness for specific actors in specific situations [26,41].

Another common principle, across societies, appears to be that the role of mental states in moral judgement depends on the nature of the violation. Across cultures, many violations of bodily purity are judged to be wrong independent of intentions ([6,33,44]; but see [45]). Moral judgements of consensual incest appear to be relatively insensitive to the beliefs of the partners in both American [6,44] and Malagasy adults [18]. Other examples of bodily violations judged with relatively strict liability include statutory rape (but not seducing a minor [33]) and ingesting taboo meats [6,24].

### (c) Why the judgement is being made

Judgements about others' behaviour are made for a variety of reasons, which can influence whether and how mental states are relevant. Public or communally assigned punishments (e.g. requirements to pay damages) may be often insensitive to intentions. Conflict resolution processes can function as truth-making institutions, to coordinate a public received opinion [46] and to uphold the authority of the legal system [33]. In these cases, there may be many valuable social functions of requiring a perpetrator to pay recompense to the victim, without publicly declaring that the perpetrator intended the harm or acted wrongly. Especially when the perpetrator and victim are both valued members of the same social group, and questions of intent are hard to establish, providing compensation without determining blame may be the best way to re-establish peace and prevent further trouble [47].

Consider, for example, the flexible use of traditional and formal justice systems among Enga communities of Papua New Guinea [48]. Traditional village courts are used to adjudicate conflicts between community members, such as marital disputes and property disputes, with the aim of restoring social cohesion. In the majority of cases, family and community contribute to cover the payment, but this is not true for repeat offenders (suggesting that intent and personal characteristics do matter, in the long run). The justice processes in these Enga communities ignore intentions when restoring social cohesion following accidents or first-time offences while turning over serious or repeated offenders to the formal justice system, which requires lawyers, witnesses and formal evidence of intent.

A similar dual system characterizes the legal and moral intuitions of American adults. The American legal system distinguishes between compensatory damages and punitive damages with regard to civil disputes. Compensatory damages are transfers of resources between the agent responsible for harm and the victim of the harm, without any imputation of malevolence or even necessarily negligence on the part of the responsible agent [40]. Americans with no legal training or expertise share the intuitions that compensatory damages should be paid to victims of accidents no matter whether anyone intended the harm [36]. Compensatory damages thus restore the victim without accusing anyone of immoral behaviour. By contrast, to express indignation and moral outrage, American juries can also assign punitive damages—for example, in tort cases, the standard for punitive damages is behaviour that displays ‘reckless indifference’.

All in all, more or less mind-minded moral judgements occur in similar contexts across societies. Moral judgements depend more on mental states when people are judging high status, competent individuals, when the violation was theft or injury, and when the purpose of the judgement is to express indignation. Moral judgements depend less on mental states when people are judging someone who is incompetent, or a whole group of people, when the action was inherently dangerous or involved a taboo about sex or food, and when the purpose of the judgement is to restore social cohesion.

We are not arguing the cultural differences in mind-minded moral judgement do not exist; the evidence described above manifestly shows such differences. The contexts that promote, or discourage, a focus on mental states may occur with different frequencies in different places. Also, important cultural differences may exist in how key abstract variables (like ‘status’, or ‘danger’) are applied to specific situations [49].

## 6. Summing up: mind-mindedness in context

Our critique of CME theories, we think, is likely to apply to many domains, not just moral judgement. Dimensions of cultural difference such as the ‘collectivist/individualist’ dimension [50] may capture some small main effects of cultural difference, but we suspect that collectivism/individualism is a parameter that can be flipped contextually within societies to a much greater degree than it varies as a main effect across societies. We may be collectivists within families, for example, but individualists at work. Similarly, we suggest that everywhere there are contexts in which one's mental states may be deemed morally irrelevant and others where they are not. Such judgements vary not just across contexts, but across individuals and time.

What we argue against, then, is thinking of mindreading as a resource that is scarce in some places and plentiful in others. Instead, we should think about it as a resource that is available everywhere, and whose use in moral judgement depends on a multiplicity of factors, including social norms but also, importantly, the reasons for which people are making judgements. Cognitive resources such as theory of mind might best be seen as ingredients that can be combined in different ways across people, places and situations. On this view, the space of moral judgements represents a mosaic of variously combined ingredients. Most of the combinations can be found just about anywhere, but appearing under different circumstances. For example, what have sometimes been characterized as unusually egalitarian sharing norms in hunter–gatherer societies might not be so different from the sharing norms seen within households in the USA [51]. Importantly, this does not mean that we should refrain from trying to understand or explain the cultural difference. What it means, in the social domain, in particular, is that our theories must include contexts and reasons for judgements as part of what we are trying to explain.

The drive for explanation pushes us to seek systematicity in complex phenomena, and this makes the search for CMEs appealing. In the case of mindreading and morality, we worry that the landscape of interactions in this domain is still not properly understood even within a single society, such as the USA. Methods and ways of conceptualizing the underlying constructs are still in their infancy, and data from applying these methods across cultures are both sparse and difficult to interpret. Before seeking to explain the cultural differences, it is important to make sure we have a proper understanding of the terrain we are trying to explain.

## Data accessibility

This article has no additional data.
